# Electrical and Optoelectronic Properties Enhancement of n-ZnO/p-GaAs Heterojunction Solar Cells via an Optimized Design for Higher Efficiency

**DOI:** 10.3390/ma15186268

**Published:** 2022-09-09

**Authors:** Lotfi Derbali

**Affiliations:** 1 Department of Physics, College of Sciences and Humanities, Shaqra University, Al Quwayiyah 19257, Saudi Arabia; derbali.lot@gmail.com or lotfi.derbali@crten.rnrt.tn; Tel.: +216-2-593-0666; 2 Laboratoire de Photovoltaique (LPV), Centre de Recherches et des Technologies de L’énergie (CRTEn), Technopole de Borj Cedria, BP: 95, Hammam Lif 2050, Tunisia

**Keywords:** effective minority carrier lifetime (τ*_eff_*), FESEM, ZnO/GaAs solar cell efficiency, microgrooved interface, ZnO nanorods, double layer anti-reflective layer

## Abstract

In this study, we report the fabrication of high quality AZO/NRs-ZnO/n-ZnO/p-GaAs heterojunction via a novel optimized design. First of all, the electrical proprieties of gallium arsenide (GaAs) substrates were enhanced via an optimized gettering treatment that was based on a variable temperature process (VTP) resulting in an obvious increase of the effective minority carrier lifetime (τ*_eff_*) from 8.3 ns to 27.6 ns, measured using time-resolved photoluminescence (TRPL). Afterward, the deposition of a zinc oxide (ZnO) emitter was optimized and examined in view of its use both as a light trapping layer (antireflection) and as the n-type partner for the p-type (GaAs) substrate. Nanorod-shaped ZnO was grown successfully on top of the emitter, as an antireflective coating (ARC), to further boost the absorption of light for a large broadband energy harvesting. The interface state of the prepared heterojunction is a key parameter to improve the prepared heterojunction performance, thus, we used laser ablation to create parallel line microgroove patterns in the GaAs front surface. We studied the effect of each step on the performance of the n-ZnO/GaAs heterojunction. The results demonstrate a significant improvement in V_oc_, J_sc_, fill factor (FF), and an obvious enhancement in the I–V characteristics, exhibiting good diode properties, giving rise to the photovoltaic conversion efficiency (η) from 8.31% to 19.7%, more than two times higher than the reference.

## 1. Introduction

Gallium arsenide (GaAs) is known as a single-crystalline material and one of the most used III-V group compound semiconductors for optoelectronic and photovoltaic devices. It is widely used in semiconductors, lasers, light emitting diodes (LEDs), heterojunction bipolar transistors (HBT’s), high electron mobility transistors (HEMT), and solar cells due to its 1.43 ev and the specific properties such as direct band gap, strong-electron-mobility, high breakdown voltage, and high thermal stability [1,2,3,4,5].

The presence of defects or impurities in this material strongly affects many of its properties [6,7,8] leading to a poor electrical and optoelectronic quality. The latter may behave as carrier traps after creating energy levels into the band gap, acting as recombination sites that reduces the minority-carrier lifetime [9,10,11], and consequently degrade the photogenerated current. The gettering process was the path to reduce or eliminate impurities, structural crystal defects, and their harmful effects by removing them from the active region to the gettering sites or trapping sites, mainly employed in silicon substrates. Therefore, the gettering of semiconductors nowadays can be an important step on the production of electronic and optoelectronic semiconductor devices, since the different gettering techniques have been reported and can be classified into external and internal gettering. The external gettering technique is based on the creation of surface gettering sites that are used mainly to increase the concentration of the trapped impurities at these sites. The latter treatment needs a careful optimization of some key parameters, investigated in our previous works, known as a variable thermal process (VTP) [9,10]. This is based on two successive annealing stages where the first should be a higher temperature annealing known as the precipitate or the diffusion temperature followed by a lower annealing temperature known as the gettering temperature.

The GaAs semiconductor has been utilized in heterojunction structures with different materials such as silicon (Si) [12], Germanium (Ge) [13], indium tin oxide (ITO) [14], graphene [15], and zinc oxide (ZnO) [16,17,18,19,20]. Actually, ZnO is exploited in photovoltaic solar cells and optoelectronic applications mainly due to its high transparency, large free-exciton binding energy, wide and direct band gap, and simple deposition [21,22]. GaAs solar cells, belonging to III-V compound semiconductors, have contributed as space and concentrator solar cells and are important as sub-cells for multi-junction solar cells. As a result of research and development, high-efficiencies [23,24] have been demonstrated with III-V compound single-junction solar cells: 29.1% for GaAs-based thin film, 24.2% for InP, 16.6% for AlGaAs, and 22% for InGaP solar cells. As many transparent conducting oxides (TCO) were used as an n-type partner for the underlying p-GaAs substrate, zinc oxide thin films is a good choice as a TCO and serves as a good emitter. Some reported works on ZnO/GaAs-based heterojunction solar cells proposed simulated models and techniques with also some experimental studies in order to improve the efficiency, but obtained results revealed limited performance [20,25]. The n-ZnO/p-GaAs heterojunction has been modeled and studied in order to improve its efficiency via a Mg-ZnO doping effect [20,26]. This study’s subject is based on an optimized experimental method that can be outlined in three steps. Firstly, a gettering process with the presence of a nano- and micro-porous structure at GaAs both surfaces that were created by vapor etching (VE) method used as trapping sites for the gettered impurities during the applied VTP process [27,28]. The latter provides GaAs samples with improved electrical properties. Thereafter, an optimized ZnO/GaAs heterojunction was prepared with a microgrooved interface to efficiently enhance the diode quality. Furthermore, utilizing a high conductive Al-doped zinc oxide (AZO) in the top of the solar cell structure has a non-negligible effect on the device performance, providing a perfect Ohmic contact [29]. In fact, many studies on ZnO/Si heterojunction solar cells have been published previously [30,31], but few experiments reported that ZnO/GaAs has better electrical quality and efficiency when optimized and designed properly, moreover, the variation of ZnO emitter parameters and especially the interface state modification on ZnO/GaAs-based solar cell performances have not been yet fully investigated. Hence, the present study is focused on integrating novel efficient methods to fabricate and analyze the device electrical properties and carrier transport in n-ZnO/p-GaAs solar cell after four steps of optimizations, yielding a combined beneficial effect, in order to obtain higher quality heterojunction performance. This technology, therefore, has the potential to be a novel high-performance, thin-film option for terrestrial photovoltaics.

The present paper is outlined as follows. Initially, the first section provides details concerning the GaAs gettering experiments and results. Afterward, the optimization of ZnO emitter deposition with various thicknesses to identify the optimum one for a higher heterojunction quality. An efficient interface engineering was introduced via a laser ablation microgrooving of the front surface of the GaAs substrates to further improve the optoelectronic properties of the processed heterojunction. Al-doped ZnO (AZO) has a higher conduction band than ZnO, faster electron mobility, and a higher electron density [32], to modify the ZnO nanorods surface in order to well restrain the recombination and promote the performance of ZnO nanorods.

## 2. Materials and Methods

**Figure 1:**
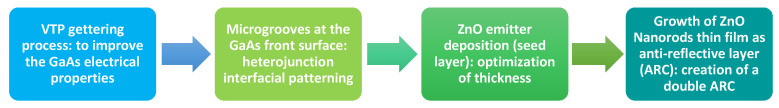
The fabrication sequence of the n-ZnO/p-GaAs heterojunction: the effect of each stage on the performance of the heterojunction was investigated. The final stage corresponds to the combination of all these effects that was carefully optimized.

The design and preparation of n-ZnO/p-GaAs heterojunction with improved electrical properties involves the steps that are shown in Figure 1: an extrinsic gettering of impurities to improve the electrical properties of the p-GaAs samples using an efficient VTP treatment, then, creation of microgrooves at the front side of the gettered GaAs sample. Subsequently, an optimized deposition of n-ZnO emitter layer is achieved with a nano-spherical-shaped morphology using the sol gel method and spin coating technique. Afterward, the synthesis of ZnO nanorods is conducted (NRs-ZnO) layer using a chemical bath deposition process.

### 2.1. Gettering of GaAs Substrates

P-GaAs (100)-doped with zinc (Zn) was used as substrate, and cut into pieces of 1 cm^2^. The GaAs substrates were initially cleaned with methanol then acetone at 40 °C. Subsequently, the wafer was thoroughly washed with de-ionized water, followed by drying under high purity N_2_ gas.

A thin nanostructured porous layer on both sides of the GaAs wafers was created by means of a vapor etching (VE) method [33,34,35]. First, the substrates were exposed to acid vapors that were issued from a mixture of HNO_3_ (65%) and HF (40%) with (1:3) concentrations at room temperature [35] for a duration of 20 min on each side of the samples. After each porous structure formation, the substrates were immediately rinsed in deionized water. Thereafter, the porous p-GaAs samples were annealed at different temperatures under (N_2_) atmosphere using an infrared furnace (IR-RTP) set-up. A total of five of these samples have been used for two different types of gettering processes that are related to the thermal annealing sequence; three samples were subjected to a thermal treatment by the classical constant temperature process (CTP) at 700 °C, 800 °C, and 900 °C for 20 min. The remaining two samples were subjected to a variable temperature process (VTP), consisting of two stages of annealing at 800 °C for 10 min then lowered to a lower temperature annealing at 600 °C and maintained for 10 min. The second sample was gettered at 900 °C for 10 min then maintained at 600 °C for 10 min. Finally, the sacrificial porous layers were removed by simple immersion of the samples in CP4 solution for few seconds and then rinsed with DI water and dried under N_2_ flow (99.99%). The CP4 solution was prepared from a mixture of the ethanoic acid CH_3_COOH, nitric acid (HNO_3_), and the hydrofluoric acid (HF) with concentration (3:5:3). The front side of the gettered samples were microgrooved using a laser ablation setup, by means of NWR 213 laser ablation system, and the main key parameters were optimized carefully: the laser ablation speed, frequency and energy, and spot size. Subsequently, in order to clean the laser micro-grooved surfaces, the substrates were immersed in HF (20%) for few seconds and then rinsed with DI water and dried under N_2_ flow (99.99%).

### 2.2. Preparation of the ZnO/GaAs Heterojunction

The performed optimization, detailed in the previous section, was of prior importance in the aim to improve the electrical properties of the p-GaAs substrates. Afterward, n-ZnO/p-GaAs heterojunction was fabricated via the sol gel method using a spin coater to deposit the n-ZnO layer. 

As the exciton is effectively divided into carriers within the electric field of the depletion zone, we chose the concentration of the base and emitter dopants in a way that allows the depletion region to be primarily created in GaAs. The latter will allow us to profit from its high absorption capabilities. According to the electric charge equilibrium equation: N_A_ W_Dp_ = N_D_ W_Dn_ [36], to obtain a relatively large depletion zone size in the base, the corresponding concentration of the dopant should be lower than the doping of the emitter. Hence, the p-type GaAs substrates were used with the minimal dopant (Zn) concentration of the order of 10^15^–10^17^cm^−3^. The deposited ZnO emitter thin films should be with a higher n-type “dopant” concentration due to the same reason.

**Figure 2:**
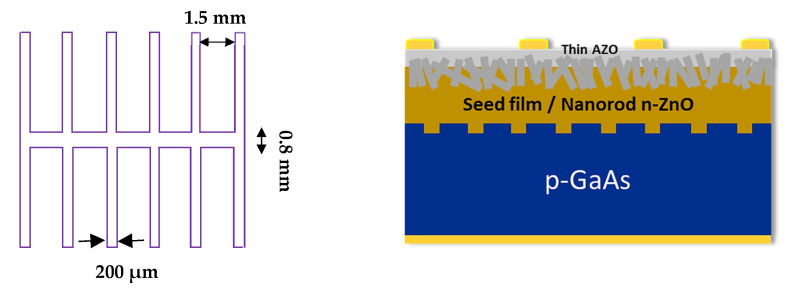
Schematic illustration of the proposed heterojunction design in its final optimized structure (**right**) and the front contact dimensions (**left**), achieved after a four-step process, detailed in this work.

Before proceeding to the ZnO thin film emitter deposition, the front surface of the gettered GaAs substrates was microgrooved using a laser ablation setup and carefully optimized on the junction interface (ZnO/GaAs), its crucial effect will be investigated in the next sections. The ZnO films were prepared through a mixture of acetate dehydrate [Zn(CH_3_COO)_2_·2H_2_O, Sigma Aldrich, St. Louis, MO, USA, purity of 98%], 2-methoxyethanol [C_3_H_8_O_2_, Aldrich], and monoethanolamine (MEA) [NH_2_CH_2_CH_2_OH, Aldrich]. The obtained zinc oxide solution (sol-gel) was deposited layer by layer on the GaAs front surface substrates and spin coated at a 2800 rpm for 10 s for each layer. After the deposition of each layer, a drying procedure was made at 150 °C for 10 s. Lastly, the deposited layers of ZnO were annealed at 400 °C for a duration of 60 min, to obtain 331 nm thickness. zinc oxide nanorods (ZnO-NRs) were grown using a hydrothermal process, performed at an atmospheric pressure and a temperature of about 70 °C [37]. Afterwards, a thin aluminum-doped zinc oxide (AZO) layer was grown onto the deposited ZnO-NRs layers by means of the atomic layer deposition (ALD) technique, to cover the top of the ZnO nanorod films, acting as a transparent electrode and an n^+^ layer to facilitate the passage of the electrons from ZnO films to the front contacts. Finally, a Ti /Au (80 nm) was evaporated as the front contacts on ZnO side, respectively, Au/Zn/Au (65 nm) contacts were formed on the rear side of GaAs substrate. Using the Kurt Lesker PVD75 e-beam evaporation technique, both the bottom and top contacts of the solar cells were deposited. As a result, heterojunction solar cells with Au/Ti/AZO/ZnO/p-GaAs/Au/Zn/Au structure were created and are illustrated in Figure 2. A series of these solar cells have been fabricated with different conditions (gettering temperature, junction interface, thickness of ZnO, and the duration of annealing) in order to choose the best conditions for electrical and photovoltaic optimization.

## 3. Results and Discussion

### 3.1. Variable Thermal Process VTP: Extrinsic Gettering of p-GaAs Substrates

**Figure 3:**
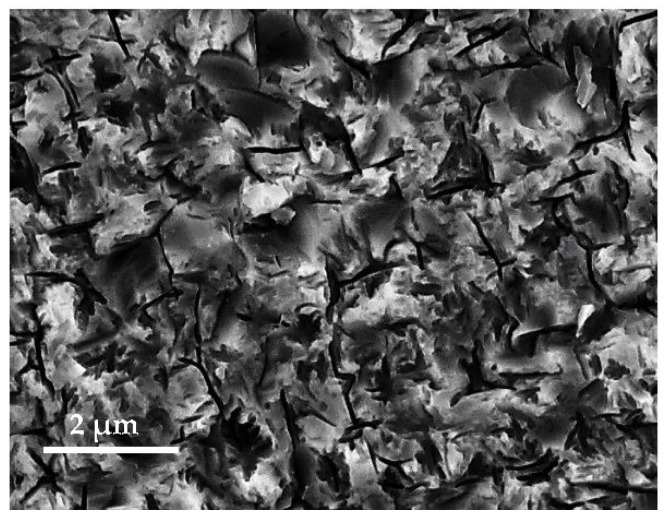
Top-view FESEM image showing the morphology of the created porous sacrificial layer, showing a nanostructured GaAs surface that was made at both sides.

The number of possible native defects and non-negligible existent impurities in a binary semiconductor such as gallium arsenide is large, as discussed in previous work [6]. The complexing of impurity atoms with native defects may also be expected. Accordingly, in GaAs-based junctions, as for solar cells applications, the main effects causing performance deterioration are the decrease of the minority carrier diffusion length and the creation of deep centers that induce recombination generation in the depletion region. The absorption of photons takes place in the first few micrometers of depth from the front surface, since gallium arsenide is a direct gap material. This makes the junction local dimensions play a crucial role and should be an important parameter determining the quality of the processed junction. In this scope, we suggest an efficient method to decrease the recombination activities via an extrinsic gettering of impurities, known to act as high active recombination centers of the photogenerated carriers with a relatively high concentration depending to the substrate type. The latter is based on the creation of sacrificial porous layers at both sides of GaAs substrates, as shown in Figure 3, which are then subjected to a variable thermal process using an RTP-IR treatment that is optimized to effectively activate the migration of a non-negligible concentration of impurities towards the sample surfaces, where the created porous sacrificial layer acts as trapping sites [34], which were removed from both surfaces after the subjected gettering process. Figure 3 is an FESEM top-view image revealing the morphology of the created sacrificial porous morphology at the GaAs surface.

The majority hall mobility (µ_H_) before and after the different applied gettering processes were carried out using the Hall effect method. The minority carrier lifetime of a direct bandgap semiconductor is typically on the nanosecond scale for the doping concentrations that were investigated. Thus, the preferred method that was adopted in this study, to perform reliable measurements, was the time-resolved photoluminescence (TRPL) method via a pulsed laser excitation using a 640 nm diode laser (PicoQuant) [38]. Both the effective minority carrier lifetime (τ*_eff_*) and µ_H_ were measured in six p-GaAs samples as a function of the gettering temperature; the results are listed in Table 1. We obtained an obvious increase of the majority carrier mobility from 138 cm^2^V^−1^s^−1^ at 25 °C (untreated substrate: reference) to 394 cm^2^V^−1^s^−1^ at 900 °C (the sample was subjected to a classical one stage thermal process (CTP)). The improvement of µ_H_ was higher in the samples that were subjected to the variable thermal process (VTP1 and VTP2), reaching the maximum value 487 cm^2^V^−1^s^−1^ at 900–600 °C (VTP2). This result can be explained by the effect of the applied gettering treatment on the decrease of recombination centers that are related in most cases to a reduction of impurities concentration, especially via the VTP2 gettering procedure that is based on a two-stages thermal process. In fact, during the subjected treatment, impurities redistribute rapidly to the GaAs surface where they will be trapped in the porous sacrificial layers. This migration has been investigated experimentally and theoretically for similar cases by many groups [39,40]. Impurity redistribution in bulk GaAs has been found to be sensitive to the annealing conditions that are used [39,41]. The out diffusion and redistribution of impurities are problematic during the preparation of the ZnO layer to fabricate the ZnO/GaAs heterojunction, since it will affect significantly the interface states, which will affect the interface quality and device performance.

In order to gain more insight in the latter mobility enhancements, the variation of the effective minority carrier lifetime (τ*_eff_*) depending on the subjected gettering procedure was investigated and represented also in Table 1. An obvious enhancement of τ*_eff_* was obtained after the classical treatment (CTP gettering process) at 700 °C, 800 °C, and 900 °C, at which the maximum value that is reached is 12.2 ns. Whereas this tendency is found to be more pronounced in samples that were subjected to a VTP process at 800–600 °C (VTP1) and 900–600 °C (VTP2). The effective minority carrier lifetime (τ*_eff_*) was increased noticeably from 8.3 ns in the reference sample to 27.6 ns in the sample VTP2. These improvements can be attributed to the crucial effect of the applied gettering treatment in sample VTP2, demonstrating that the used two stages gettering treatment (variable thermal process VTP) is more efficient to getter the GaAs substrates to improve its electrical properties. The measured τ*_eff_* are in good agreement with the obtained mobility (µ_H_) results.

n-ZnO/p-GaAs heterojunctions were fabricated using the above gettered GaAs substrates in order to further investigate their effect on the electrical properties of the processed heterojunctions. In this first stage, the thickness of the n-ZnO emitter layer is 302 nm.

**Figure 4:**
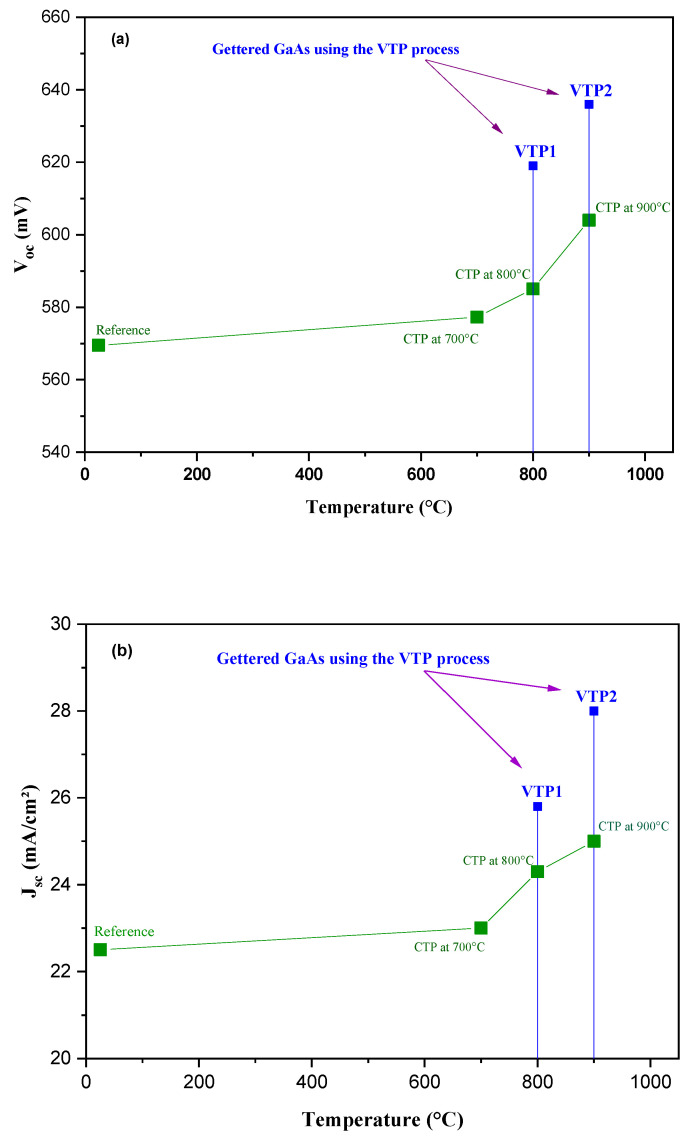
(**a**) Dependency of the open circuit voltage (V_oc_) and (**b**) the short circuit current density (J_sc_) with the gettering temperatures during the infrared (IR) rapid thermal process (RTP), proving the crucial effect when applying the VTP route on the electrical properties of ZnO/p-GaAs heterojunction, compared to the classical one stage process (CTP).

The measured open circuit voltage (V_oc_) and short-circuit current density (J_sc_) are summarized in Figure 4a,b. The obtained results show an obvious dependency of V_oc_ and J_sc_ with temperature, reaching the highest value for a two stage gettering process at 800–600 °C (VTP1) and 900–600 °C (VTP2). These increments are related to the subjected gettering treatment of the GaAs substrates, after the decrease of the carrier recombination activities. The latter is found to be in good agreement with the obtained variations of µ_H_ and τ*_eff_*. During the gettering treatment, the thermal stress that is applied on the GaAs substrates leads to the migration of the existent impurities from the crystalline volume and a diffusion towards the preferential damaged surface (the sacrificial porous layers) at both sides of the GaAs. The applied variable thermal process (VTP) can noticeably enhance the efficiency of the subjected gettering, and with optimized parameters and process, it can reduce the size of metal-precipitates [42] and, therefore, effectively activate the migration towards the surfaces and increase the rate of impurities that can reach the surfaces without affecting the quality of the GaAs substrate and causing its degradation. V_oc_ and J_sc_ are affected by the presence of different types of impurities, especially the iron ones; the V_oc_ have a strong correlation with the concentration of these impurities: the decrease of the iron impurities causes a direct increase in the V_oc_ values [43]. The obtained results in both VTP1 and VTP2 prove that, compared to the classical thermal process CTP (i.e., one stage: 700 °C, 800 °C, 900 °C), the two-stages annealing (VTP) provided a higher gettering efficiency. This process has been used to avoid the stress that is induced during the heat treatment for the long-term and consequently prevents the appearance of other defects. Some previous works [10,42] noticed that a careful optimization of the thermal treatment is necessary.

### 3.2. Introducing Microgrooves at the ZnO/GaAs Heterojunction Interface (Using the Gettered Substrate)

**Figure 5:**
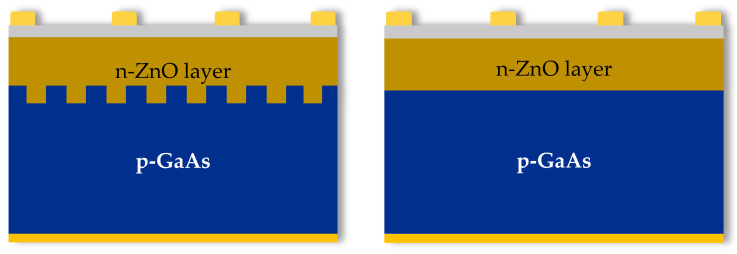
Schematic drawings of the investigated solar cell’s structure with (**left**) and without (**right**) microgrooves at the interfaces.

Figure 5 illustrates schemas of the investigated n-ZnO/p-GaAs solar cells without and with interface patterning. In the proposed design, we suggest the use of a modified ZnO/p-GaAs interface with microgrooves to obtain a higher photogeneration of carriers, particularly at this region since the created microgrooves will augment the dimension of the active surface at the interface, resulting in a significant enhancement of the heterojunction optoelectronic performance. The processed substrates at this stage are gettered substrates VTP2, that were found to possess the best electrical properties as discussed in the previous section.

**Figure 6:**
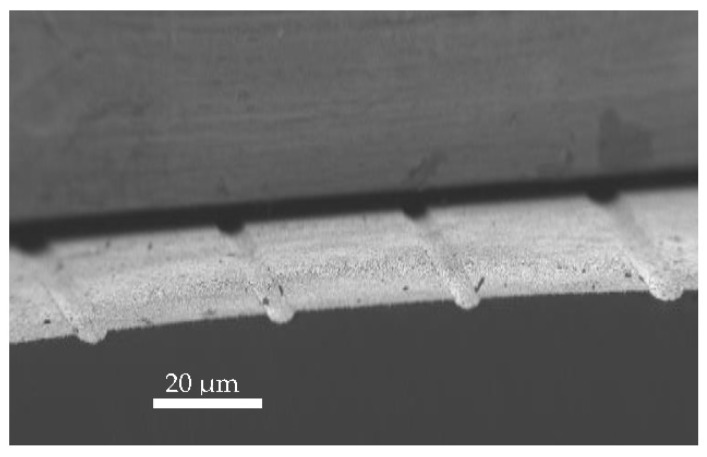
Typical cross-sectional FESEM image of GaAs substrate.

Figure 6 shows FESEM cross-section image of the gettered GaAs substrate. The FESEM image clearly shows microgrooves that were produced by laser ablation setup on the GaAs surface. The sample size is 1cm with a distance around 40 μm between the microgrooved lines as shown in the image, so the total number of the parallel lines patterns on the entire surface was 250.

**Figure 7:**
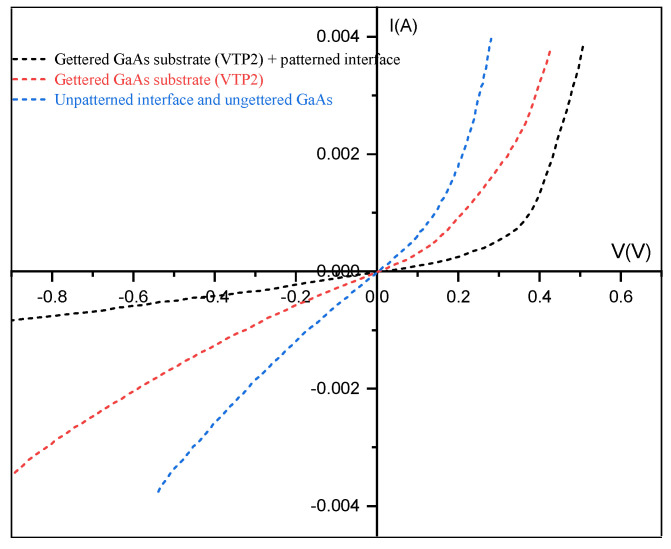
The I-V characteristics that were measured for three heterojunctions: Reference sample without any treatment, sample without microgrooves but gettered GaAs substrate, sample that was prepared with a gettered GaAs substrate (VTP2) and microgrooved interface.

We used the gettered GaAs samples VTP2, to fabricate the ZnO/GaAs heterojunctions, since they exhibit the best electrical properties compared to the other treatments as detailed. The latter experiments are based firstly on a systematic comparison, as shown in Figure 7, between the measured current-voltage characteristics in the dark in the ZnO/GaAs heterojunction with and without microgrooves at the interfaces. In the proposed design, we suggest an interfacial modification via the creation of microgrooves at the heterojunction interfaces, to study its role on the electrical properties enhancement of the heterojunction as a result of the improved quality of the depletion region. 

The recorded I-V response in Figure 7, clearly points out that the heterojunction that was prepared with a gettered GaAs substrate (VTP2) and microgrooved at the interface shows significantly higher dark and undoubtedly higher photocurrent values, compared with the ZnO/GaAs heterojunction with a flat interface even with or without using the gettered GaAs substrate. Due to the reduction of the impurity concentration in the gettered GaAs, the leakage current decreased significantly after the subjected treatment VTP2. The I-V characteristics clearly show a significant improvement of the rectifying behavior and a noticeable decrease of the reverse current in the heterojunction that was fabricated with the gettered GaAs substrate and containing microgrooves at the interface. These improvements that were observed in dark I-V characteristic are mainly related to the increased specific surface area of the microgrooved interface of the heterojunction, while the photocurrent is higher both due to the increased specific surface area, which results in the generation of more photocarriers in the conduction band, and higher absorption in the microgrooved interface due to multiple reflections of the incident light. Moreover, the deposited ZnO emitter leads to the reduction of natural oxide bonds, such as Ga-O and As-O, which contribute to high defect density on GaAs surfaces.

### 3.3. Optimization of the ZnO Emitter Thickness

**Figure 8:**
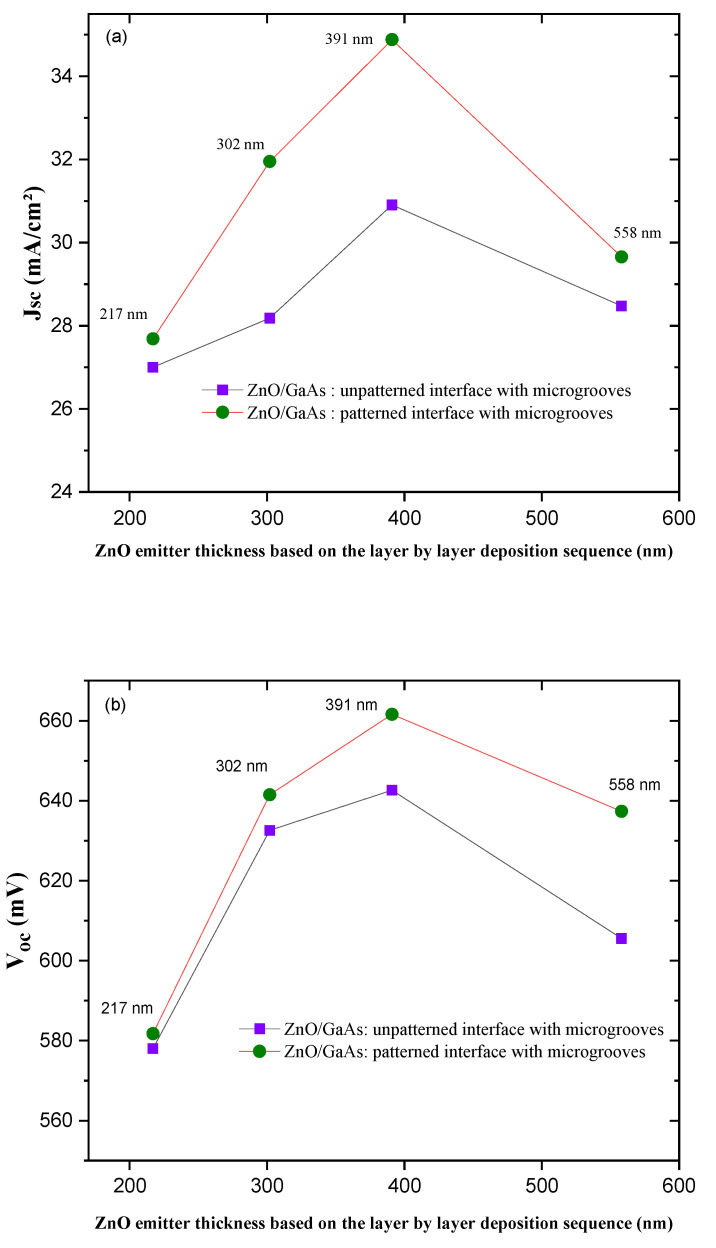
The effect of ZnO emitter thickness on the (**a**) J_sc_ and (**b**) V_oc_, highlighting the vital role of microgrooves at the heterojunction interface.

To further enhance the built-in potential of the heterojunction, and thus achieve even better device performance, we investigated the effect of ZnO emitter thickness by varying the number of the deposited layers during the preparation of the ZnO/GaAs heterojunction. Accordingly, we studied the effect of the ZnO emitter thickness by varying the preparation sequence. Thereby, different thicknesses were grown onto the microgrooved front surface of the gettered p-GaAs (samples that were subjected to the VTP2 gettering process) in order to identify the optimal emitter thickness that provides further improvements in the heterojunction electrical properties. The variation effect of the ZnO emitter thickness is presented in Figure 8.

As expected, there is an obvious dependency of the measured V_oc_ and J_sc_ with the ZnO thickness, as shown in Figure 8. The increment of J_sc_ and V_oc_ values can be related to the increased light absorption in the ZnO thin films and the improvement of the photo-generated current in the ZnO/GaAs heterojunction solar cell. Increasing the thickness of the emitter (total thickness n-ZnO and AZO) from 302 nm to 391 µm revealed further enhancements of the heterojunction’s electrical properties. The latter can be attributed to a narrow depletion zone that was created due to the lowest gradient of dopant concentrations between the n-p partners in the case of thickness that is inferior to 391 nm. Most likely, this thickness dependence is attributed to the result of the absorption edge inception of the overlaying ZnO thin films that contributes to the short wavelengths absorption, which in turn decreases the amount of light that can be caught by the effectively absorbing GaAs surface region. As clearly seen in Figure 8, exceeding 391 nm of thickness results in an obvious degradation of the V_oc_ and J_sc_ values, probably due, in some cases, to an increased series resistance effect [44], lower quality of the created junction and a decreased photogeneration rate. Henceforth, we will consider the optimum obtained thickness that was pointed out in Figure 8 (391 nm that was prepared via a sequence of spin coated 12 layers).

**Figure 9:**
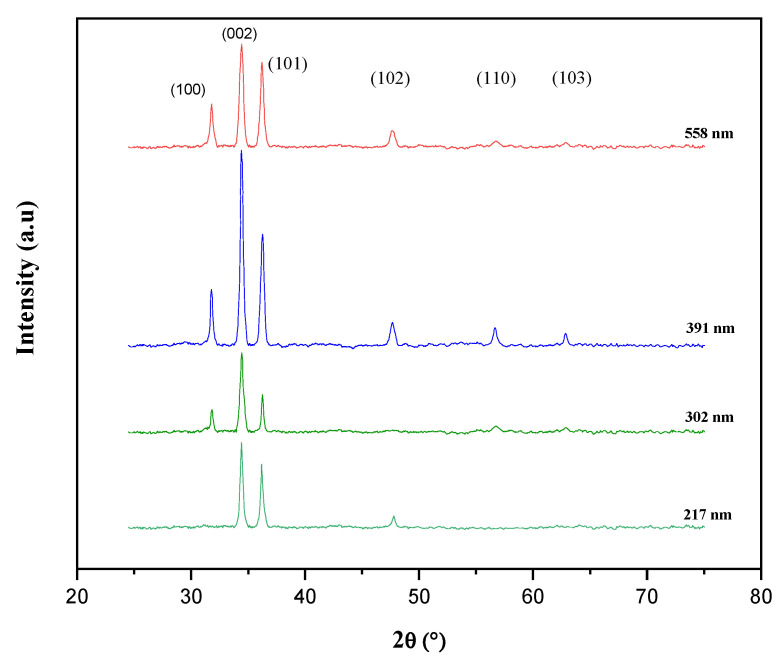
The XRD spectra of the deposited ZnO emitter thin films: thickness optimization.

We have further examined the possible enhancement of the crystallinity in the 302 nm after being increased to 391 nm emitter thickness. Figure 9 shows the XRD spectra of the ZnO thin films at both thicknesses. As shown in the figure, the (100), (002), (101), (102), (110), (103), and (112) peaks were observed. All the diffraction peaks were located similarly to the standard ZnO sample (JCPDS Card No: 36-1451) in the recorded range 2θ, indicating that deposited ZnO thin film has a hexagonal wurtzite structure. The recorded XRD spectra revealed a preferential orientation in the (002) direction, in the 391 nm thickness, indicating an improved crystallinity that was confirmed by the increased intensity of the recorded spectra. Whereas, in the 217 nm and 558 nm, the crystallites orientation can be described as randomly oriented, without a highly dominant orientation and inferior intensities.

As can be seen from the recorded peaks, the deposited ZnO layer with 391 nm thickness shows an increase in intensity and crystallinity, compared to the sample with 302 nm and the other thicknesses, which can be related to an apparent crystallinity enhancement in the 391 nm ZnO layer. Moreover, the intensity of the (002) peak is stronger than the other peaks, signifying that ZnO crystallites are highly oriented with the c-axis. Table 2 lists the corresponding (002) peak structural parameters, such as the crystallite size (D), the dislocation density (δ), and the strain (ɛ) values [45], for both XRD patterns of the ZnO thin films with thicknesses of 302 nm and 391 nm, since they showed the best electrical and structural properties compared to the other samples.

For the most intense peak, the crystallite size (D) was determined by Scherrer’s equation:

(1) $$D=\frac{0.9\;\lambda }{\beta \;\cos\theta \;}\;$$

where β is the full width at half of the peak maximum (FWHM), λ is the wavelength of the incident X-ray, and θ is Bragg’s diffraction angle. The density of dislocations and strain values are determined by:

(2) $$\delta =\frac{1}{D^{2}}\;$$

And

(3) $$\varepsilon =\frac{\beta }{4\;\tan\;\theta }$$

The mean crystallite size (D) increased from 20.4 nm to 28 nm. On the other hand, the dislocation density and the strain values were decreased. It is worth noting that the increase in the crystallite size improved the quality of the crystal [46,47]. The dislocation density reveals the imperfection in the crystalline lattice [48], and its decrease in the deposited 391 nm is in good agreement with the detailed optimization in the last section that is related to the electrical property’s enhancements in the deposited ZnO emitter for 391 nm thickness.

As the microstructural quality of the deposited ZnO thin films has a vital effect on its electrical properties, we can characterize the disorder in the films via the slope of Urbach tail or Urbach energy (*E_u_*). Moreover, the latter can provide further evaluation of the defect concentration in the deposited thin films. As crystal disorders increase, the electronic transitions will arise from the filled valence band to the energy levels of the defects, instead of electronic transitions from the filled valence band to the empty conduction band. In fact, Urbach energy has the dimension of energy which is related to the width of the localized states in the band gap. It is considered as a valuable parameter which is closely related to the disorder in the film crystalline lattice and is expressed as [49]:

(4) $$\alpha ={\;\alpha }_{0}.\exp\left(\frac{h\nu }{E_{u}}\right)$$

where *α*_0_ is a constant and *E_u_* is the Urbach energy or disorder energy.

Hence, *E_u_* could be determined through the plot of ln (α) versus photon energy (hυ). Low Urbach energy means a lower density of impurities and defects in the ZnO thin film network, while high values or increased trend of *E_u_* can be attributed to high defects and impurities that are related mainly to oxygen vacancies in the prepared thin films [50,51]. The obtained values are 118 mev in 302 nm ZnO emitter thickness and 87.2 mev in 391 nm thickness, emphasizing an obvious decrease in E_u_ that is suggested to imply the reduction of defects, or else disorder, proving the higher quality of the prepared thin film with 391 nm thickness. The latter confirms the clear correlation with our findings that are related to the discussed structural properties and the obtained electrical measurements.

### 3.4. ZnO Nanorods Growth as ARC

**Figure 10:**
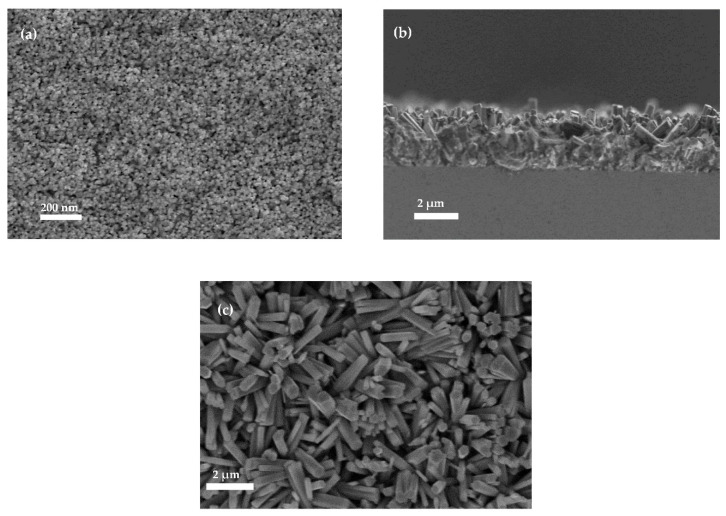
(**a**) Top view of the optimized ZnO emitter (391 nm thickness), (**b**) SEM cross-section view showing the NRs-ZnO/n-ZnO/p-GaAs heterojunction, and (**c**) top view of the grown ZnO nanorods (NRs ZnO).

ZnO nanorods were grown directly on the ZnO emitter surface as an efficient anti-reflective coating (ARC), aiming to further boost the light absorption at the front side of the final processed n-ZnO/p-GaAs heterojunction the solar cell. The latter significantly improved the absorption of the incidental photons by the ZnO nanorods layer and increased the diffusion and the trapping of light via multiple reflections that were produced inside the internal structure of the nanorods. Shown in Figure 10 are the FESEM images revealing a cross section view of the heterojunction after the deposition of the ZnO nanorods antireflective coating (a), and a top view after covering the total emitter by AZO thin film (b). It is worth noting that deposited AZO onto the front surface of the emitter has excellent optical transmission performance, with a high donor concentration allowing for a superior conductivity of the emitter [52,53].

**Figure 11:**
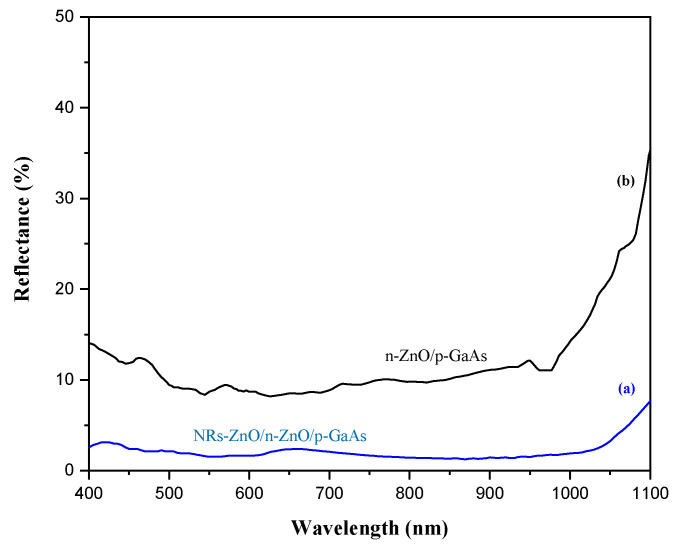
Reflectance spectra of the prepared heterojunction solar cells with (a) the presence of the nanorods ZnO layer grown on the emitter ZnO thin film, to form a double layer antireflective coating; and without (b).

The presence of such nanostructures (NRs-ZnO), shown in Figure 10a,b, has a crucial role in the optical properties since it largely reduces the reflection losses at the front surface of the heterojunction solar cell, which results in an improved absorbance of solar irradiance and higher electron-hole pair photogeneration rate. We used the UV-VIS-NIR spectroscopy to study the total reflectivity of the resulting double layer antireflective layers: the ZnO emitter and the grown ZnO nanorods. The comparison was made between heterojunctions with and without the presence of the NRs-ZnO layer. Both samples show a significant decrease of their front surface total reflectivity within the investigated wavelength range of 400 nm–1100 nm. As depicted in Figure 11, the presence of NRs-ZnO allowed the lowest reflection that was reduced to around 3%, while the heterojunction without the presence of NRs-ZnO exhibits a higher reflection around 10% within a limited wavelength range. Accordingly, the proposed double layer antireflective coating proves its vital effect to minimize the front surface reflectance allowing to further boost the carrier photogeneration.

**Figure 12:**
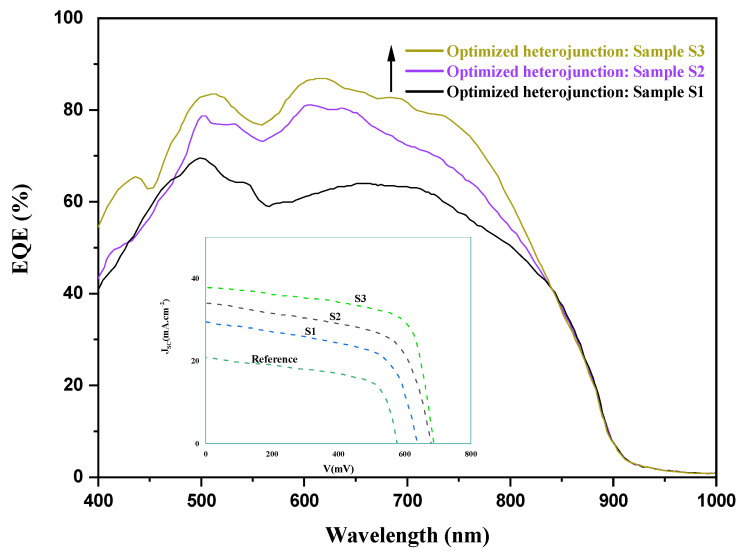
The external quantum efficiency (EQE) spectra of n-ZnO/p-GaAs heterojunction solar cells: (S1) heterojunction with a gettered GaAs substrate via the VTP2 process and an optimized emitter thickness of 391 nm, (S2) same as S1 with the incorporation of microgrooves at the heterojunction interface, (S3) same as S2 with the introduction of ZnO nanorods at the front surface of the emitter to boost the absorption at the front side of the device. The illuminated J-V characteristics are shown in the inset.

The efficiency of the solar cell tells us what percentage of the total energy that is carried by the photons can be transformed into electrical power. Particularly, we focused our interest in quantum efficiency, known as the ratio of the collected carriers’ number by the solar cell to the number of photons of a given energy incident on the device [34]. Measurements were performed utilizing Bentham PVE300 system, between 400 and 1000 nm spectral region. The results of the external quantum efficiency (EQE) in the prepared samples through each fabrication process can be seen in Figure 12.

The EQE spectra shows a comparison between three curves for three samples (S_1_, S_2_, S_3_), where the first sample (S_1_) represents a heterojunction with a gettered VTP2 substrate and an optimized emitter thickness, with a flat interface state. Microgrooves were introduced in the (S_2_) sample. In sample (S_3_), both the microgrooves at the interfaces and ZnO nanorods (NRs) at the front surface of the emitter were introduced. The EQE improvement is quite significant after introducing the microgrooves at the heterojunction interface in sample S2, which is estimated to be about 22% at 610 nm. The response of the solar cell S_3_ found to be clearly the highest improvement in the entire spectral region with a maximum enhancement of 30% measured at 610 nm. The shape of the EQE curves results from the layered structure of the solar cells and the reflection that can occur between the layers interfaces [26].

The main PV action that occurs takes place mostly within the GaAs substrate interface, which was our intention that was expressed during the planning phase and the introduction of the microgrooves. Accordingly, within the wavelength range between 500 and 800 nm, the EQE exhibits a high response, particularly in the heterojunction structure with microgrooves (sample S2 and S3). Microgrooves on the GaAs front surface can increase the absorption and significantly decrease the reflectivity of light at the ZnO/GaAs interface, demonstrating a vital effect on the reduction of the reflectivity as judged from the important enhancement in the device performance [54]. The latter will have a crucial role in the increase of the carriers photogeneration in that region of the heterojunction. From the present results, the ZnO emitter thickness and morphology revealed a crucial role on the heterojunction quality. Further improvements were obtained in sample S3 in the presence of ZnO nanorods that were introduced to boost the absorption of incident light. In the presence of ZnO nanorods, the GaAs solar cell showed a broad-range EQE enhancement from 400 to around 800 nm and there was a 30% maximum enhancement in the EQE.

As depicted in Table 3, we can clearly see the beneficial effect of each optimized step, and its vital outcome when combined to boost the performance of the n-ZnO/p-GaAs heterojunction (heterojunction S3). The V_OC_ of the cell was raised by 104 mV in S3 compared to the reference cell, J_sc_ was increased from 22.8 mA·cm^−2^ to 35.8 mA·cm^−2^, and the fill factor (FF) was improved from 65.6% in the reference cell to 77.2% in S3. Accordingly, the patterned interface in the prepared heterojunction obviously led to significant electrical properties. The latter reveals that the introduction of microgrooves at the heterojunction interface can effectively increase the carrier photogeneration at the depletion region, which was confirmed with the EQE study. The conversion efficiency (η) of the proposed heterojunction design (heterojunction S3) is found to be improved from 8.31% to 19.7%, as shown in Table 3.

## 4. Conclusions

In summary, a significant enhancement in the n-ZnO/p-GaAs solar cell performance was achieved as a combined effect of three optimized factors: subjecting the GaAs substrate to an external gettering, optimizing the ZnO emitter thickness; introducing microgrooves at the interface to influence the optoelectronic properties which is an important region of the device; and finally the growth of a thin ZnO nanorods film to boost the absorption of incident light at the front side of the heterojunction solar cell. A careful optimization was conducted at each stage to limit and identify the key experimental parameters. Firstly, at the first stage, we successfully improved the electrical properties of the GaAs substrate via an efficient external gettering based on a VTP process utilizing a rapid thermal process (RTP), optimized carefully to increase the minority carrier lifetime (τ*_eff_*) and the majority carrier mobility (µ_H_) from 8.3 ns, in the reference GaAs substrate, to 27.6 ns after gettering via the described VTP2 process, and from 138 cm^2^V^−1^s^−1^ to 487 cm^2^V^−1^s^−1^, respectively, by employing the transient photoluminescence technique (TRPL). The key parameters short circuit current density (J_sc_), open circuit voltage (V_oc_), fill factor (FF), and the external quantum efficiency (EQE) after each stage in the fabrication of the final device are compared. The photovoltaic conversion efficiency was enhanced more than two times compared to the reference heterojunction from 8.31 to 19.7. However, a deep insight in the patterned interface state and the recombination mechanisms and types in the AZO/NRs-ZnO/n-ZnO/p-GaAs heterojunction requires a detailed examination and further optimizations in order to achieve possible higher efficiencies.

## Figures and Tables

**Table 1:** The effective minority carrier lifetime (τ_eff_) and the majority hall mobility (µ_H_) in p-GaAs before (untreated reference) and after the gettering process as a function of temperature.

		CTP Process (One Stage Classical Annealing)			VTP Process (Two-Stages Annealing)	
	**Reference (Untreated)**	**700 °C**	**800 °C**	**900 °C**	**800–600 °C (VTP1)**	**900–600 °C (VTP2)**
**µ_H_ (cm^2^V^−1^s^−1^)**	138	331	368	394	420	487
**τ_eff_ (ns)**	8.3	10.6	12.2	11.8	19.4	27.6

**Table 2:** Structural parameters of the grown ZnO thin films onto the p-GaAs substrates.

Thickness	2θ (°)	FHWM (Radian)	D (nm)	$\delta \;\left(\times 10^{14}\;Lines/m²\right)$	ε (%)
**302 nm**	34.39	0.41	20.4	23.9	57.4
**391 nm**	34.40	0.28	28	12.2	38.6

**Table 3:** Photovoltaic parameters for the investigated heterojunction solar cells: (S_1_) represents a heterojunction that is fabricated using GaAs gettered substrate (VTP2) and an optimized ZnO emitter thickness (391 nm), with a flat interface state. Microgrooves at the interface were added in (S_2_). In sample (S_3_), both the microgrooves at the interfaces and ZnO nanorods at the front surface of the ZnO emitter were introduced.

Solar Cell	J_sc_ (mA·cm^−2^)	V_oc_ (mV)	FF	η (%)
Reference ZnO/GaAs	22.6	576	65.6	8.31
S1	30.8	637.8	69.4	13.4
S2	34.6	679.3	75.1	17.22
S3	36.8	683	78.4	19.7

## Data Availability

The data that are presented in this study are available on request from the corresponding author.
